# Acute Octodrine (DMHA) Exposure Disrupts Cardiac Function, Locomotor Activity, and Neuronal Activity-Responsive Transcription in Zebrafish Larvae

**DOI:** 10.3390/ijms27198560

**Published:** 2026-09-24

**Authors:** Eduardo Kennedy Carrão-Dantas, Ouwais Aljabasini, Eva Prats, Israel Felzenszwalb, Carlos Fernando Araujo-Lima, Demetrio Raldúa

**Affiliations:** 1 Laboratory of Environmental Mutagenesis, Department of Biophysics and Biometry, Rio de Janeiro State University (UERJ), Rio de Janeiro 20551-030, RJ, Brazil; uerj.felzen@gmail.com (I.F.); araujo.lima@unirio.br (C.F.A.-L.); 2 Integrated Laboratory of Environmental Mutagenesis, Department of Genetics and Molecular Biology, Federal University of Rio de Janeiro State (UNIRIO), Rio de Janeiro 20211-010, RJ, Brazil; 3 Institute for Environmental Assessment and Water Research (IDAEA-CSIC), Jordi Girona, 18, 08034 Barcelona, Spain; ouwais.aljabasini@idaea.csic.es; 4 Research and Development Center (CID-CSIC), Jordi Girona, 18, 08034 Barcelona, Spain; eva.prats@cid.csic.es

**Keywords:** octodrine, DMHA, zebrafish, neurobehavior, cardiotoxicity, atrioventricular block, immediate early genes, dietary supplements

## Abstract

Octodrine (2-amino-6-methylheptane; DMHA) is a sympathomimetic stimulant detected in sports-performance and weight-loss supplements despite limited toxicological characterization and regulatory concerns. Here, we used 7-day-post-fertilization zebrafish larvae to characterize the acute cardiac, neurobehavioral, and transcriptional effects of octodrine after 2 h of exposure. Cardiac responses were biphasic: 5 µM octodrine significantly increased atrial and ventricular rates, whereas concentrations ≥ 150 µM produced progressive cardiac depression and higher concentrations disrupted atrioventricular conduction. At 450 µM, all larvae showed complete absence of detectable cardiac activity; mortality was not independently assessed. Modelling of the inhibitory phase yielded IC_50_ values of 195.6 µM for atrial rate and 188.8 µM for ventricular rate. At substantially lower concentrations, octodrine significantly reduced spontaneous locomotor activity. After Holm correction across all 24 planned time-by-treatment comparisons, a transient effect remained detectable at 50 nM, with more sustained hypoactivity at 0.5 and 5 µM across most of the 2 h exposure period. At the transcriptional level, 3.5 µM octodrine significantly reduced *fosab* and *egr1* expression, whereas *fkbp5* was induced at selected concentrations; *npas4a* showed a significant overall treatment effect but no dose-specific comparison remained significant after blocked analysis and multiplicity correction. *nr4a1* was significantly downregulated at 350 µM, whereas *th1* and *kcnh2a* expression was not significantly altered. Together, these findings show that neurobehavioral/transcriptional responses and pronounced cardiac dysfunction occurred over different concentration ranges under the conditions tested. The results provide an initial in vivo hazard characterization of octodrine and support further investigation of its neuropharmacological mechanisms, internal exposure, and cardiovascular safety.

## 1. Introduction

The expanding market for pre-workout, thermogenic, weight-loss, and performance-enhancing supplements has increased human exposure to pharmacologically active stimulants for which efficacy and safety are often incompletely characterized. In addition to conventional ingredients such as caffeine, analytical surveys have repeatedly identified synthetic or experimental sympathomimetic compounds in commercially available products, sometimes under ambiguous ingredient names or with substantial discrepancies between labelled and measured contents [1,2]. This is of particular concern in sports supplements, where stimulant combinations may result in poorly predictable pharmacological interactions and may also expose athletes to substances prohibited in competition. Among the compounds that have emerged in this context is octodrine, also known as 2-amino-6-methylheptane or 1,5-dimethylhexylamine (DMHA).

Octodrine is not a newly synthesized compound. It was pharmacologically investigated during the mid-twentieth century and used in pharmaceutical preparations, including nasal decongestant formulations, before largely disappearing from medical use. Decades later, it re-emerged as an ingredient in pre-workout and weight-loss supplements, frequently promoted as an alternative to other sympathomimetic stimulants such as 1,3-dimethylamylamine (DMAA) [3]. Chemical analyses have confirmed the presence of octodrine in commercial sports and weight-loss supplements. Octodrine, together with other experimental or prohibited stimulants, has been identified in products marketed in the United States [1], while this compound has been found in several commercial supplements, with reported amounts reaching 112 mg per serving [4]. The latter study also found a racemic enantiomeric distribution and synthetic by-products, supporting a synthetic rather than botanical origin of the octodrine detected in these products. Regulatory concerns have consequently increased: the U.S. Food and Drug Administration considers DMHA an unsafe food additive that does not meet the statutory definition of a dietary ingredient and considers dietary supplements containing it to be adulterated [5]. Octodrine is also classified by the World Anti-Doping Agency as a specified stimulant prohibited in competition [6].

Despite this pattern of human exposure, the pharmacology and toxicology of octodrine remain remarkably poorly characterized. Much of the experimental evidence originates from studies conducted more than seven decades ago. In the classical study by Fellows (1947) [7], 2-amino-6-methylheptane displayed pressor activity and increased cardiac rate and contractile amplitude in anesthetized dogs, together with local anesthetic effects, whereas central nervous system stimulation was minimal in rats at non-toxic doses. Other early studies similarly described cardiovascular and respiratory actions, but these investigations predated modern molecular and neurobehavioral approaches [3]. More recent literature has focused predominantly on the occurrence of octodrine in supplements, analytical detection, regulatory issues, and doping rather than on experimental characterization of its biological effects. Although octodrine is generally marketed and described as a stimulant and catecholaminergic actions have been proposed, its molecular targets and the contribution of dopaminergic, adrenergic, or other neuronal pathways remain poorly resolved. Likewise, contemporary experimental information on its potential cardiotoxicity is extremely limited. This lack of mechanistic toxicological information contrasts with both its availability in commercial products and the cardiovascular adverse effects associated with sympathomimetic stimulant exposure.

Zebrafish provide a particularly suitable vertebrate model for addressing this knowledge gap because neurobehavioral and cardiovascular effects can be evaluated within the same intact organism over short exposure periods. Spontaneous locomotion provides an integrated functional readout of central and sensorimotor activity, whereas heart rate and atrioventricular conduction provide complementary physiological endpoints that can reveal direct or systemic cardiovascular effects. Evaluating these domains in the same developmental stage is particularly useful for determining whether locomotor changes occur at concentrations below those that compromise cardiac function, thereby reducing the likelihood that altered movement simply reflects severe cardiovascular impairment. Early-life zebrafish possess functional monoaminergic systems and exhibit quantifiable locomotor responses to psychoactive compounds, while their optical accessibility permits direct assessment of atrial and ventricular activity and atrioventricular conduction [8,9]. This combination has proven useful for the integrated characterization of stimulant compounds. For example, synthetic cathinones and classical psychostimulants produce compound-specific alterations in locomotor activity, cardiac rate, rhythmicity, and atrioventricular conduction in zebrafish, demonstrating that neurobehavioral and cardiac effects can be resolved within a common experimental framework [9,10]. The model further enables functional phenotypes to be linked with rapid molecular responses, including changes in genes associated with monoaminergic signalling, neuronal activity, cellular stress, and cardiac electrophysiology.

In the present study, we therefore used 7-day-post-fertilization (dpf) zebrafish larvae to provide an integrated characterization of the acute effects of octodrine across neurobehavioral, cardiac, and molecular levels. Spontaneous locomotor activity was assessed as a sensitive functional readout of neuroactive effects, whereas high-speed cardiac imaging was used to quantify atrial and ventricular rate and atrioventricular conduction. To explore molecular responses associated with these phenotypes, we additionally examined the expression of genes involved in dopamine synthesis and transport (*th1* and *slc6a3*), neuronal activity-dependent transcription (*fosab*, *egr1*, *npas4a*, and *nr4a1*), stress signalling (*fkbp5*), and cardiac repolarization (*kcnh2a*). By integrating these complementary endpoints across concentration ranges, the study aimed to characterize the acute neurobehavioral, cardiac, and molecular effects of octodrine and to identify responses that may help guide subsequent mechanistic investigation.

## 2. Results

### 2.1. Acute Octodrine Exposure Impairs Cardiac Function and Atrioventricular Conduction

At 450 µM octodrine, no detectable atrial or ventricular activity was observed in any larva during the cardiac recordings. Because no independent viability assay was performed, this endpoint is described here as cardiac arrest rather than mortality. The 450 µM group was excluded from quantitative heart-rate GEE and inhibitory concentration–response modelling but was retained in the categorical cardiac-status analysis. Among the 0–350 µM groups, octodrine produced a biphasic effect on cardiac activity (Figure 1A; individual data are available in Supplementary Dataset S1). Generalized estimating equations (GEE) analysis revealed a strong overall effect of octodrine concentration on heart rate (Wald χ^2^(5) = 2286.91, *p* < 0.001), as well as a significant concentration × cardiac chamber interaction (Wald χ^2^(3) = 12.39, *p* = 0.006), whereas no significant effect of experimental replicate was detected (Wald χ^2^(2) = 3.61, *p* = 0.165). At 5 µM, atrial and ventricular rates were significantly increased relative to the corresponding controls (both Holm-adjusted *p* < 0.001). The numerical reduction observed at 50 µM did not reach statistical significance (both Holm-adjusted *p* = 0.076). From 150 µM onward, octodrine produced a marked and progressive reduction in both atrial and ventricular rates (all Holm-adjusted *p* < 0.001). Full GEE model statistics, estimated marginal means, and planned contrasts are provided in Supplementary Table S1.

Because the cardiac response was biphasic, a single sigmoidal model was not fitted across the complete concentration range. Instead, the inhibitory phase was analyzed separately after normalizing each heart-rate value to the mean vehicle-control rate from the corresponding experimental replicate and cardiac chamber. The stimulatory 5 µM group and the 450 µM cardiac-arrest group were excluded, and 50, 150, 250, and 350 µM were fitted to a constrained inhibitory logistic model with the upper and lower asymptotes fixed at 100% and 0%, respectively. The resulting IC_50_ was 195.6 µM for atrial rate (95% CI: 179.4–211.7 µM; Hill slope = 2.40; R^2^ = 0.732) and 188.8 µM for ventricular rate (95% CI: 174.1–203.5 µM; Hill slope = 2.58; R^2^ = 0.761). Model specifications, residuals, and fitted curves are provided in Supplementary Table S2 and Supplementary Figures S1 and S2, and the underlying calculations are included in Supplementary Dataset S1.

Octodrine also altered cardiac conduction/status at the higher concentrations tested (Figure 1B,C). All larvae in the control, 5, 50, and 150 µM groups showed 1:1 atrioventricular conduction. At 250 µM, 22/24 larvae retained 1:1 conduction and 2/24 displayed a 2:1 pattern. At 350 µM, 16/24 larvae showed 1:1 conduction, 3/24 showed 2:1 conduction, 2/24 showed >2:1 conduction, and 3/24 exhibited cardiac arrest. At 450 µM, all 24 larvae exhibited cardiac arrest. In the proportional-odds ordinal logistic model, concentration was coded as an ordered rank from 0 to 6 (control, 5, 50, 150, 250, 350, and 450 µM) and experimental replicate was included as a covariate. The model converged and showed a strong concentration-dependent shift toward more severe categories (likelihood-ratio χ^2^(1) = 143.88, *p* < 0.0001; concentration coefficient β = 4.486; odds ratio = 88.74 per one-rank increase, 95% CI: 15.37–512.41), whereas the overall contribution of experimental replicate did not reach significance (likelihood-ratio χ^2^(2) = 5.84, *p* = 0.054). Because the 450 µM group was necessarily assayed on a separate plate, an ordinal sensitivity analysis excluding this group was also performed and retained a strong concentration effect (likelihood-ratio χ^2^(1) = 31.25, *p* < 0.0001; odds ratio = 11.71, 95% CI: 2.39–57.32). A complementary binary sensitivity analysis of any cardiac impairment versus 1:1 conduction, also excluding 450 µM, likewise supported the concentration trend (odds ratio = 9.27, 95% CI: 2.08–41.24; *p* = 0.0034). The A/V ratio is retained in Figure 1C as a descriptive measure only for larvae with detectable atrial and ventricular activity; no ratio was calculated for 0/0 recordings. Full cardiac-status summaries, model diagnostics, and sensitivity analyses are provided in Supplementary Table S3 and Supplementary Dataset S1.

### 2.2. Octodrine Reduces Spontaneous Locomotor Activity

Octodrine exposure significantly altered spontaneous locomotor activity during the 2 h exposure period (Figure 2; individual data are available in Supplementary Dataset S1). The prespecified GEE analysis revealed significant effects of treatment (Wald χ^2^(3) = 41.37, *p* < 0.001) and time interval (Wald χ^2^(7) = 593.65, *p* < 0.001), together with a significant treatment × time interaction (Wald χ^2^(21) = 44.87, *p* = 0.002). A highly significant experimental-replicate effect was also present (Wald χ^2^(2) = 46.59, *p* < 0.0001), reflecting substantial differences in baseline locomotor activity among the three independent experiments. In an expanded sensitivity model that additionally included treatment × experimental replicate, this interaction was significant (Wald χ^2^(6) = 27.57, *p* = 0.000113), indicating that the magnitude of treatment effects differed among experiments. Experiment-specific model-derived 2 h treatment/control ratios nevertheless confirmed the direction of the 5 µM effect in all three experiments: 32.5% of control (95% CI: 20.2–52.2), 58.7% (95% CI: 47.8–72.1), and 70.4% (95% CI: 52.3–94.8) in experiments 1–3, respectively. Thus, the 95% confidence interval remained below 100% in each experiment despite significant between-run heterogeneity. Experiment-stratified descriptive summaries and all model-derived sensitivity estimates are provided in Supplementary Table S4 and Supplementary Dataset S1.

To address multiplicity across the full set of planned comparisons, Holm correction was applied across all 24 treatment-versus-control contrasts (three treatments × eight intervals). Under this more conservative correction, the 50 nM group differed significantly from the vehicle only during the 15–30 min interval (*p* = 0.039), indicating a transient low-concentration effect. At 500 nM, significant reductions were retained at 15–30, 45–60, 60–75, and 90–105 min (Holm-adjusted *p* ≤ 0.033). The 5 µM group showed the most sustained effect, with significantly reduced locomotor activity during each of the first seven intervals (0–105 min; Holm-adjusted *p* ≤ 0.0064), whereas the final 105–120 min interval was not significant (*p* = 0.180). Full raw and globally Holm-adjusted *p* values are provided in Supplementary Table S4 and Supplementary Dataset S1.

### 2.3. Octodrine Modulates Neuronal Activity- and Stress-Responsive Gene Expression

Exposure to octodrine for 2 h produced concentration-related changes in the transcriptional profile of genes associated with dopaminergic function, neuronal activity, stress signalling, and cardiac electrophysiology (Figure 3; individual Cp, ΔCt, and efficiency-corrected expression values are available in Supplementary Dataset S1). Because one biological pool per condition was obtained from each of eight plates, the analysis accounted for plate as a blocking factor. The reference gene *ppiaa* remained stable across treatments in the blocked model (treatment: *F*(4, 28) = 0.323, *p* = 0.860; plate: *F*(7, 28) = 0.595, *p* = 0.754). Blocked analyses revealed significant treatment effects on *slc6a3* (*F*(4, 28) = 3.56, *p* = 0.018), *fosab* (*F*(4, 28) = 8.60, *p* < 0.001), *egr1* (*F*(4, 28) = 15.43, *p* < 0.0001), *npas4a* (Friedman *Q*(4) = 16.30, *p* = 0.0026), *nr4a1* (*F*(4, 28) = 25.06, *p* < 0.0001), and *fkbp5* (*F*(4, 28) = 33.12, *p* < 0.0001); whereas, no significant overall treatment effects were detected for *th1* (*F*(4, 28) = 0.88, *p* = 0.489) or *kcnh2a* (*F*(4, 28) = 2.17, *p* = 0.098). No significant plate effect was detected for any gene analyzed parametrically (all *p* ≥ 0.103). Full blocked-model and post hoc statistics are provided in Supplementary Table S5.

At 0.35 µM, transcriptional effects were limited to a modest but significant induction of *fkbp5* (Holm-adjusted *p* = 0.013). At 3.5 µM, *fosab* (*p* = 0.000538) and *egr1* (*p* = 0.000223) were significantly downregulated, while *fkbp5* was induced (*p* = 0.0000928). Although *npas4a* showed a significant overall treatment effect, none of its individual treatment-versus-control comparisons remained significant after the paired blocked analysis and Holm correction (all adjusted *p* ≥ 0.156).

At 35 µM, no individual treatment-versus-control comparison remained significant after Holm adjustment; the *nr4a1* comparison was borderline (adjusted *p* = 0.052). At 350 µM, *fosab* (*p* = 0.0315), *egr1* (*p* < 0.0001), and *nr4a1* (*p* < 0.0001) were significantly downregulated, whereas *fkbp5* was strongly induced (*p* < 0.0001). Although the overall treatment effect for *slc6a3* was significant, none of the individual octodrine concentrations differed significantly from the vehicle control after Holm adjustment. Similarly, *th1* and *kcnh2a* showed no significant dose-specific changes.

## 3. Discussion

The present study provides an integrated in vivo characterization of the acute effects of octodrine across behavioural, cardiac, and molecular endpoints. Three main findings emerge. First, spontaneous locomotor activity was reduced at submicromolar-to-low-micromolar concentrations, although the 50 nM effect was transient after correction across the complete family of planned comparisons. Second, a similar low-micromolar exposure range was associated with reduced expression of several neuronal activity-responsive transcripts, while the dopaminergic synthesis and transport markers *th1* and *slc6a3* were comparatively preserved. Third, at higher concentrations, octodrine caused pronounced bradycardia and atrioventricular conduction abnormalities. These observations indicate different concentration ranges for neurobehavioral/transcriptional and pronounced cardiac effects under the conditions tested, while the incomplete overlap between the concentration series precludes defining sharply separated toxicological thresholds. This distinction remains relevant because octodrine is pharmacologically under-characterized despite its documented presence at substantial doses in sports and weight-loss supplements and continuing regulatory concern [1,3,4,5].

The behavioural data indicate that neural function is affected by acute octodrine exposure. After correction across all 24 planned time-by-treatment comparisons, the 50 nM effect was limited to the 15–30 min interval, whereas 500 nM produced effects at several intervals and 5 µM produced sustained hypoactivity through 105 min. Locomotor activity also progressively decreased over the recording period in vehicle-control larvae. Such time-dependent reductions in spontaneous locomotion following transfer to a behavioural recording environment have previously been described in zebrafish larvae and interpreted as habituation to the testing apparatus [11,12]. In the present study, recording was intentionally initiated immediately after addition of the exposure solutions and placement of the plate in the DanioVision chamber so that the onset and evolution of the acute response could be followed from the beginning of exposure; consequently, no pre-exposure acclimation period was included. Time and treatment × time were explicitly modelled in the GEE. Nevertheless, the pronounced temporal decline in controls and the significant treatment × experimental-replicate interaction indicate that baseline state and experimental run influenced response magnitude, and a standardized acclimation comparison would be valuable in future studies. Notably, the cardiac response observed at 5 µM consisted of increased rather than depressed atrial and ventricular rates, while pronounced bradycardia and conduction abnormalities required substantially higher concentrations. This argues against the hypoactivity at 5 µM being a secondary consequence of depressed cardiac function and supports a direct or indirect neuropharmacological contribution.

The transcriptional profile provides additional evidence that neural function is affected within the same low-micromolar concentration range. The genes examined here represent complementary activity-responsive transcriptional markers in zebrafish. *fosab*, the zebrafish orthologue of mammalian c-FOS, is widely used as a molecular readout integrating recent neuronal activity in the larval brain [13], whereas *egr1* is an immediate-early gene rapidly induced by sensory stimulation in zebrafish larvae [14]. Similarly, *npas4a* is predominantly expressed in the zebrafish brain and is strongly induced by neuronal activation [15], while *nr4a1* has been identified among neuronal activity-dependent immediate-early genes associated with neuroplastic responses in the zebrafish brain [16]. In the blocked analysis, *fosab* and *egr1* were significantly reduced at 3.5 µM, whereas *npas4a* showed a significant overall treatment effect without a dose-specific comparison surviving multiplicity correction. The low-micromolar transcriptional profile therefore remains consistent with altered neuronal activity, but the evidence for a specific *npas4a* change at 3.5 µM is weaker than initially inferred. A related transcriptional pattern has been reported in zebrafish following developmental exposure to amphetamine, nicotine, and oxycodone, all of which produced downregulation of *fosab*, *egr1*, and *npas4a* together with alterations in locomotor behaviour [17]. Although the exposure paradigms differ substantially, these findings support the interpretation that modulation of neuronal activity-responsive genes can accompany drug-induced neurobehavioral alterations in zebrafish. Acute modulation of the same transcriptional network has also been observed following 2 h exposure to the synthetic cathinone MDPV in 3 dpf zebrafish embryos, although with a markedly different concentration-dependent profile [18]. In that study, *fosab* and *egr1* were upregulated at higher MDPV concentrations, whereas *npas4a* and *nr4a1* showed biphasic regulation, including downregulation at lower concentrations and induction at the highest concentration tested [18]. Thus, rapid modulation of neuronal activity-responsive genes appears to be a recurrent feature of acute stimulant exposure in zebrafish, while the direction and magnitude of the response may depend on compound identity, concentration, and developmental stage.

In contrast, *th1* expression was not significantly altered, while *slc6a3* showed only a modest overall concentration effect, with no individual octodrine concentration differing significantly from the vehicle control. These findings suggest that the acute response does not involve major transcriptional remodelling of dopamine synthesis or dopamine transporter expression within the 2 h exposure window. Importantly, this does not exclude acute effects of octodrine on dopaminergic neurotransmission through changes in transporter function, dopamine release, receptor activation, or other non-transcriptional mechanisms, none of which can be inferred from whole-larva transcript abundance.

The induction of *fkbp5* provides an additional indication that octodrine engages stress-responsive signalling. In zebrafish, *fkbp5* is a well-characterized glucocorticoid receptor (GR)-responsive gene that participates in feedback regulation of glucocorticoid signalling. Its expression exhibits GR-dependent regulation, and genetic disruption of GR signalling markedly alters *fkbp5* transcription in zebrafish [19,20]. Interestingly, *fkbp5* was also significantly induced following a 2 h exposure to MDPV in zebrafish embryos, providing an additional precedent for rapid engagement of stress-responsive transcription by structurally distinct stimulant compounds [18]. The induction observed following low-micromolar octodrine exposure is therefore compatible with engagement of glucocorticoid-responsive signalling and may reflect activation of the hypothalamic–pituitary–interrenal stress axis. However, *fkbp5* expression alone cannot establish either increased cortisol production or activation of the HPI axis, and these possibilities should be tested directly. At 350 µM, the interpretation must be even more conservative since cardiac function is already severely compromised. Transcriptional alterations at this concentration may consequently reflect generalized physiological stress, altered tissue oxygenation, or systemic toxicity rather than a primary neuronal response. Measurements of whole-body cortisol, monoamine concentrations, and spatially resolved neuronal activity would help distinguish these mechanisms.

Cardiac responses to octodrine were biphasic. At 5 µM, atrial and ventricular rates were modestly but significantly increased, whereas concentrations ≥ 150 µM produced a progressive reduction in cardiac rate. Because the initial stimulatory phase violates the monotonicity assumption of a conventional concentration–response curve, quantitative modelling was restricted to the inhibitory phase (50–350 µM), yielding IC_50_ values of approximately 196 µM for the atrium and 189 µM for the ventricle. Cardiac conduction/status also deteriorated at the upper end of the concentration range, with 2:1 and >2:1 conduction patterns and cardiac arrest appearing at 250–450 µM. Zebrafish are particularly sensitive to pharmacological perturbation of cardiac repolarization, and compounds that interfere with repolarizing currents can produce pronounced bradycardia and AV block [21]. More recent functional studies have further demonstrated that both ERG potassium-channel blockade and inhibition of T-type calcium channels can produce bradycardia and AV conduction block in zebrafish larvae through different electrophysiological mechanisms [22]. Thus, the cardiac phenotype observed here is compatible with disruption of cardiac excitability or conduction but does not, by itself, identify the underlying molecular target.

In this context, the absence of significant changes in *kcnh2a* expression should be interpreted cautiously. Although human cardiac IKr is mediated by KCNH2/hERG, the corresponding repolarizing current in the zebrafish heart is predominantly mediated by the paralogue Kcnh6a rather than Kcnh2a [22]. Therefore, unchanged *kcnh2a* transcript abundance cannot be used to infer preservation of ERG-mediated repolarization. Moreover, direct pharmacological inhibition of ion-channel function would not necessarily require transcriptional modulation. Interestingly, acute MDPV exposure in zebrafish also produces marked bradycardia and AV block without significant transcriptional modulation of *kcnh6a* or *kcnq1* [18], further illustrating the limitations of transcript abundance as a surrogate for acute cardiac ion-channel function. Direct electrophysiological or pharmacological studies, together with assessment of *kcnh6a* and other relevant cardiac ion-channel targets, would therefore be required to determine the mechanism underlying octodrine-induced conduction abnormalities.

The toxicological relevance of these findings lies primarily in hazard identification rather than direct prediction of human risk. Analyses of commercially available supplements have detected approximately 72 mg of octodrine per serving, more than twice the highest historical pharmaceutical dose, while another survey reported concentrations reaching 112 mg per serving [1,4]. These findings demonstrate that consumers may be exposed to pharmacologically substantial quantities of octodrine despite the limited information available regarding its safety. Nevertheless, nominal waterborne concentrations in zebrafish cannot be directly translated into human doses or circulating concentrations. Zebrafish larvae can absorb, metabolize, accumulate, and excrete xenobiotics to markedly different extents depending on compound-specific physicochemical and metabolic properties, such that nominal bath concentration may differ substantially from internal exposure [23]. Determination of octodrine uptake, internal concentrations, and metabolite profiles would therefore be necessary before quantitative comparisons with human exposure can be attempted. The same consideration applies to establishing whether the low-micromolar concentrations associated with behavioural effects in the present study are achievable in human plasma or target tissues.

Several limitations should therefore be considered. The study evaluated only acute exposure and a relatively small molecular panel, and transcript measurements were performed in whole larvae, limiting both anatomical and cell-type resolution. The experiments did not directly quantify internal octodrine concentrations, monoamines, cortisol, neuronal activity, or cardiac electrophysiology. Spontaneous locomotor activity is an integrated but non-specific neurobehavioral endpoint. The behavioural assay used distance travelled as its primary functional endpoint and did not include complementary behavioural domains such as startle responsiveness, thigmotaxis, or sensorimotor gating. Recording began without a separate acclimation period, and treatment positions in the cardiac, behavioural, and qRT-PCR assays were systematic rather than randomized or rotated, so positional effects cannot be separated completely from treatment effects. In the cardiac assay, the 450 µM group was necessarily located on a second plate and was therefore completely confounded with plate identity; importantly, this group was excluded from quantitative heart-rate GEE and inhibitory concentration–response modelling, and sensitivity analyses excluding 450 µM confirmed the concentration-dependent cardiac-status effect. The qRT-PCR samples were generated in a single exposure run distributed across eight plates; all five treatments were represented on every plate, and plate was included as a blocking factor to account for any potential shared plate-level variation, but treatment rows were fixed across plates and between-run reproducibility of the transcriptional findings was not assessed. In addition, although 1% DMSO was kept constant in all exposure groups and vehicle controls and acute exposure at this concentration has been reported not to alter larval locomotion [24], a water-only control was not included; therefore, subtle solvent effects or solvent-by-octodrine interactions cannot be excluded, particularly for behavioural and stress-responsive endpoints. Finally, the significant treatment × experimental-replicate interaction in the locomotor sensitivity analysis indicates between-run heterogeneity in effect magnitude. Gene-expression measurements provide information on transcriptional responses but cannot establish rapid pharmacological interactions with neurotransmitter transporters, receptors, or ion channels. Despite these limitations, the convergence between locomotor suppression and reduced expression of neuronal activity-responsive genes within the low-micromolar range, together with pronounced cardiac effects at higher concentrations, provides a coherent initial hazard characterization of this poorly studied stimulant used in dietary supplements and identifies specific neuroendocrine and electrophysiological mechanisms warranting further investigation. These zebrafish findings should not be interpreted as direct evidence of human risk.

## 4. Materials and Methods

### 4.1. Zebrafish Maintenance and Embryo Production

Adult wild-type short-fin sexually mature zebrafish, within the typical standard length range for breeding stock (3.0–3.5 cm), were obtained from a commercial supplier (Exopet, Madrid, Spain) and were maintained under a 12 h:12 h light/dark cycle and a temperature of 28 ± 1 °C at the Research and Development Center (CID-CSIC) in a recirculating water system (Aquaneering Inc., San Diego, CA, USA).

Embryos were obtained through natural spawning in breeding tanks from groups composed of three females and two males. Fertilized eggs were collected within 30 min post-spawning (lights-on). Only high-quality embryos at the 50% epiboly stage were selected under a stereomicroscope (Nikon SMZ1500, Champigny-sur-Marne, France). Embryos were transferred to a crystallizing dish containing embryo water [Milli-Q water with 90 mg/L Instant Ocean (Aquarium Systems, Sarrebourg, France) and 0.58 mM CaSO_4_·2H_2_O, pH 6.5–7.0, 750–900 µS/cm conductivity] and incubated at 28 ± 1 °C in a climatic chamber (POL-EKO APARATURA KK350, Wodzisław Śląski, Poland) under a 12 h:12 h light/dark cycle until the start of the experiments at 7 dpf.

All procedures were approved by the Institutional Animal Care and Use Committees at the CID-CSIC and conducted in accordance with the institutional guidelines under a licence from the local government (agreement number 11336).

### 4.2. Chemicals

Octodrine (2-amino-6-methylheptane; molecular formula C8H19N; molecular weight 129.24 g/mol; CAS 543-82-8) was purchased as an analytical standard from Sigma-Aldrich (St. Louis, MO, USA). Octodrine is not currently used as an approved clinical drug; historically, it was marketed in Europe in multi-ingredient pharmaceutical products at doses of 8–33 mg, whereas contemporary supplement analyses have reported approximately 72 mg and up to 112 mg per serving [1,4]. The waterborne concentration ranges used here were not selected as clinical dose equivalents. Rather, they were chosen as endpoint-specific hazard-characterization ranges informed by preliminary range finding and the sparse historical/modern literature: nanomolar-to-low-micromolar concentrations were used to detect neurobehavioral effects, whereas higher micromolar concentrations were required to characterize cardiac rate and conduction. Because nominal bath concentrations cannot be directly converted to human administered doses or plasma concentrations, no quantitative human-dose equivalence is inferred [23]. Stock solutions (50 mM) were freshly prepared in DMSO on the day of each experiment. Working solutions were prepared by dilution in embryo water and contained a final DMSO concentration of 1% (*v*/*v*) in all exposure groups; the corresponding vehicle control also contained 1% DMSO. This common vehicle concentration was maintained across treatments and endpoints to avoid concentration-dependent differences in solvent exposure and to accommodate the highest octodrine concentration tested (450 µM), which required 0.9% (*v*/*v*) of the 50 mM DMSO stock solution. Although 1% DMSO is at the upper end of concentrations commonly used as a vehicle in zebrafish assays, acute exposures at this concentration have been reported to produce no detectable alterations in larval locomotor behaviour [24]. The pH of all exposure solutions, including the 1% DMSO vehicle-control medium, was adjusted during preparation to approximately 8.3 with 1 M NaHCO_3_. This value was selected as a compromise between approaching the pKa of octodrine (pKa = 10.42) and maintaining exposure conditions within a pH range physiologically tolerated by zebrafish larvae [25]. Final pH after the 2 h exposure was not recorded; this is acknowledged as a methodological limitation.

### 4.3. Cardiac Assessment

The cardiac assessment followed the protocol described elsewhere [18]. In each of three independent experiments, 7 dpf larvae were exposed for 2 h to vehicle control or 5, 50, 150, 250, 350, or 450 µM octodrine (8 larvae per group per experiment; 24 larvae per group overall; one larva per well; 1 mL solution). Two 48-well plates were required per independent experiment. On Plate 1, vehicle control and 5, 50, 150, 250, and 350 µM groups occupied rows A–F, respectively, using wells 1–8 in each row. The 450 µM group occupied wells A1–A8 of Plate 2. Treatment positions were kept constant rather than rotated across the three independent experiments, for a total of six plates. Consequently, treatment and row position could not be separated within Plate 1, and the 450 µM group was additionally completely confounded with plate identity. All groups included in the quantitative heart-rate GEE and inhibitory concentration–response analysis (control through 350 µM) were nevertheless located on the same plate within each experiment. Each larva/well was treated as the experimental unit, while experimental replicate was incorporated into the statistical models to account for batch-level variation. To minimize movement during imaging, larvae were briefly anesthetized (0.08 mg/mL tricaine methane-sulfonate [MS-222], 10 s) and embedded in 3% methylcellulose on a depression microscope slide. Cardiac activity was recorded laterally with a high-speed camera (ace U acA1440-220 um, Basler AG, Ahrensburg, Germany) attached to a stereomicroscope (SMZ1500, Nikon, Champigny-sur-Marne, France) at 100 fps for 20 s using Pylon Viewer 64-Bit software (9.1.0.1614) at 28 ± 1 °C.

Atrial and ventricular beat frequencies were quantified using DanioScope v1.0 (Noldus Information Technology, Wageningen, The Netherlands), which derives heart rate from pixel-intensity fluctuations over time [26]. Atrial and ventricular activity were analyzed separately. When both chambers displayed measurable activity, the atrioventricular (A/V) ratio was calculated as atrial rate divided by ventricular rate. Cardiac conduction/status was then categorized as 1:1 (A/V < 1.5), 2:1 (1.5 ≤ A/V < 2.5), or >2:1 (A/V ≥ 2.5). Recordings with both atrial and ventricular rates equal to zero were classified as cardiac arrest and no A/V ratio was calculated. At 450 µM, all larvae showed complete absence of detectable atrial and ventricular activity. Because sustained absence of heartbeat is an established indicator of lethality in vertebrates and an accepted apical lethality endpoint in zebrafish toxicity testing, this finding is consistent with a lethal effect. However, no independent post-exposure viability assessment was performed to confirm irreversibility.

The categorical cardiac-status analysis was used for inferential assessment of conduction impairment because A/V ratios were strongly concentrated at 1.0 and undefined for 0/0 recordings. The continuous A/V ratio was retained only as a descriptive endpoint among larvae with measurable atrial and ventricular activity.

Cardiac-status frequencies and descriptive A/V ratios are provided in Supplementary Table S3, and individual-level classifications and ratios are provided in Supplementary Dataset S1.

Because the overall cardiac response was biphasic, nonlinear modelling was restricted a priori to the inhibitory phase. For each larva, atrial and ventricular rates were normalized to the mean vehicle-control rate from the corresponding experimental replicate and chamber (vehicle = 100%). The 5 µM group was excluded because it represented the initial stimulatory phase, and the 450 µM group was excluded because all larvae showed cardiac arrest. Data from the 50, 150, 250, and 350 µM groups were fitted separately for atrial and ventricular responses to Y = 100/[1 + (X/IC_50_)^h^] using nonlinear least-squares regression, where Y is heart rate expressed as a percentage of the corresponding vehicle control, X is the octodrine concentration, IC_50_ is the concentration producing 50% inhibition, and h is the Hill slope. IC_50_ values, Hill slopes, 95% confidence intervals, R^2^, and individual residuals are provided in Supplementary Table S2 and Supplementary Dataset S1; fitted curves and residual plots are shown in Supplementary Figures S1 and S2.

### 4.4. Behavioural Assessment

Basal locomotor activity (BLA) was assessed in 7 dpf zebrafish larvae exposed for 2 h to 50 nM, 500 nM, and 5 μM octodrine. Vehicle control larvae were exposed to embryo water containing 1% DMSO. Three independent experiments were performed, with 12 larvae per treatment in each experiment (n = 36 larvae per group overall; one larva per well). Treatments were assigned systematically by plate columns rather than by randomized well allocation. The individual larva/well was considered the experimental unit, and experimental replicate was included in the statistical analysis. Recordings were made on the DanioVision platform (Noldus Information Technology, Wageningen, The Netherlands) under near-infrared illumination with the temperature maintained at 28 °C. Video tracking was performed using EthoVision XT software (v13 for recording, v16 for analysis).

Immediately after addition of the exposure solutions, the plate was transferred to the DanioVision chamber and recording was initiated without a prior acclimation period. This design was chosen to follow locomotor activity from the beginning of the acute exposure rather than after a separate pre-exposure stabilization period. Locomotor behaviour was quantified during eight consecutive 15 min observation windows over a 120 min period (0–15, 15–30, 30–45, 45–60, 60–75, 75–90, 90–105, and 105–120 min). For each larva, BLA was defined as the total distance (cm) travelled within each window [18]. Distance travelled was the prespecified behavioural endpoint; complementary behavioural domains were not recorded in this experiment.

### 4.5. RNA Preparation and qRT-PCR Analysis

Total RNA was extracted from pools of six 7 dpf zebrafish exposed for 2 h to vehicle control or 0.35, 3.5, 35, or 350 μM octodrine using TRIzol Reagent (Invitrogen, Carlsbad, CA, USA). Gene-expression samples were obtained in a single exposure run using eight 48-well plates. On every plate, vehicle control, 0.35, 3.5, 35, and 350 µM groups occupied rows A–E, respectively, using wells 1–6 in each row; these treatment positions were kept constant across the eight plates. Each plate therefore contained 30 larvae, and the six larvae belonging to a given condition on each plate were pooled to generate one biological sample. This yielded eight biological pools per concentration (*n* = 8; 48 larvae per concentration; 240 larvae in total), with one pool per condition from each plate. RNA concentration and purity were determined spectrophotometrically using a NanoDrop ND-8000 spectrophotometer (NanoDrop Technologies, Wilmington, DE, USA). RNA integrity was assessed using an Agilent 2100 Bioanalyzer (Agilent Technologies, Santa Clara, CA, USA), with RNA Integrity Number (RIN) values ranging from 9 to 10. Following DNase I treatment (Ambion, Austin, TX, USA), 1 μg of total RNA was reverse-transcribed using the First Strand cDNA Synthesis Kit (Roche Diagnostics, Mannheim, Germany) with oligo(dT), according to the manufacturer’s instructions.

Quantitative real-time PCR (qRT-PCR) was performed using a LightCycler 480 System (Roche Diagnostics, Mannheim, Germany) and SYBR Green Master Mix (Roche Diagnostics, Mannheim, Germany). The amplification protocol consisted of an initial incubation at 95 °C for 15 min, followed by 45 cycles of 95 °C for 10 s and 60 °C for 30 s. Each experimental condition comprised eight biological pools, one from each plate, with three technical replicates analyzed for each biological sample. Primers targeting *th1*, *slc6a3*, *fosab*, *egr1*, *npas4a*, *nr4a1*, *fkbp5*, and *kcnh2a* were designed using Primer-BLAST (National Center for Biotechnology Information, Bethesda, MD, USA; accessed on 24 July 2026), which incorporates Primer3 version 2.5.0 for primer design. Primer efficiency and specificity were verified prior to analysis. The amplification efficiency factors (E) were 1.915 for *th1*, 1.891 for *slc6a3*, 1.971 for *fosab*, 1.990 for *egr1*, 1.950 for *npas4a*, 1.975 for *nr4a1*, 2.033 for *fkbp5*, 2.008 for *kcnh2a*, and 1.922 for *ppiaa*. No amplification was detected in either no-template controls (NTCs) or no-reverse-transcription controls (no-RT controls). Specificity of the qRT-PCR products was confirmed by melt-curve analysis, which yielded a single amplification-product peak for each target gene. Primer sequences for these genes, together with those for the reference gene *ppiaa* (peptidyl-prolyl isomerase A), are provided in Supplementary Table S6. Individual Cp values for *ppiaa* and all target genes are provided in the Gene Expression sheet of Supplementary Dataset S1.

Gene-expression values were normalized against the reference gene *ppiaa*. Because the measured amplification-efficiency factors differed modestly from 2.0, graphical relative-expression values were recalculated using the target-specific and *ppiaa* efficiencies rather than the uncorrected 2^−ΔΔCt^ approximation. For each biological sample, log_2_ relative expression was calculated as (mean vehicle-control Cp_target − sample Cp_target) × log_2_(E_target) − (mean vehicle-control Cp_*ppiaa* − sample Cp_*ppiaa*) × log_2_(E_*ppiaa*). Values are therefore expressed as efficiency-corrected log_2_ fold change relative to the vehicle-control group. Recalculation changed the mean log_2_ fold-change estimates only minimally (maximum absolute difference from the original 2^−ΔΔCt^ representation = 0.052), but the efficiency-corrected values are used throughout the revised Figure 3 and Supplementary Dataset S1. Statistical inference was performed on individual ΔCt values rather than fold-change values.

### 4.6. Data Analysis

Statistical analyses were performed using IBM SPSS Statistics version 32.0.0.0 Statistics and GraphPad Prism version 10.0 (GraphPad Software, San Diego, CA, USA), as specified below. Statistical significance was set at *p* < 0.05.

Atrial and ventricular heart rates were analyzed jointly using generalized estimating equations (GEEs), with octodrine concentration and cardiac chamber as fixed factors, their interaction included in the model, and experimental replicate entered as a blocking factor. Larva identity was specified as the subject variable, with atrial and ventricular measurements treated as repeated observations within each subject. GEE was selected to account for the within-larva correlation between cardiac chambers while providing robust inference in the presence of heteroscedasticity. A normal distribution with identity link, robust covariance estimator, and unstructured working correlation matrix were used. Planned comparisons between each octodrine concentration and the corresponding vehicle control were adjusted for multiple testing using the sequential Bonferroni (Holm) procedure.

The inhibitory-phase cardiac concentration–response analysis was performed as described in Section 4.3 after normalization to the corresponding experiment-specific vehicle control; 5 and 450 µM were excluded from this fit, and the resulting parameter is reported as IC_50_ rather than EC_50_.

Inferential analysis of AV conduction was based on the ordered cardiac-status categories (1:1, 2:1, >2:1, cardiac arrest) rather than on the continuous A/V ratio. A cumulative-logit proportional-odds ordinal regression was fitted with concentration coded as an ordered rank (0 = control, 1 = 5 µM, 2 = 50 µM, 3 = 150 µM, 4 = 250 µM, 5 = 350 µM, 6 = 450 µM) and experimental replicate included as indicator covariates. Model convergence was verified from the optimizer output. The proportional-odds assumption was explored using threshold-specific cumulative-logit models; the first threshold was estimable, whereas the higher thresholds were non-estimable because severe outcomes were sparse and separated by concentration, precluding a reliable formal Brant-type test. Robustness was therefore assessed using two additional sensitivity analyses excluding the separately plated 450 µM group: a second ordinal model and a binary logistic model comparing any cardiac impairment (2:1, >2:1, or cardiac arrest) with 1:1 conduction. The A/V ratio itself was summarized descriptively only for larvae with measurable atrial and ventricular activity.

Locomotor activity was measured repeatedly in the same larvae over time, resulting in correlated within-subject observations that violate the independence assumption of conventional between-group tests. Therefore, locomotor activity data were analyzed using GEE to account for repeated measurements from the same larva across eight consecutive 15 min intervals. Distance travelled was modelled using a gamma distribution with a log link because the data were strictly positive and right-skewed. Larva identity was specified as the subject variable and time interval as the repeated factor. Octodrine treatment, time interval, and their interaction were included as fixed effects, while experimental replicate was included as a fixed blocking factor. An AR(1) working correlation structure and robust sandwich covariance estimator were used. Estimated marginal means were calculated on the original response scale. Planned comparisons between each octodrine treatment and vehicle control within each interval were adjusted using a single sequential Bonferroni (Holm) procedure across the complete family of 24 planned contrasts (3 treatments × 8 intervals). To assess reproducibility across independent experiments, a sensitivity GEE additionally included the treatment × experimental-replicate interaction; experiment-stratified cumulative-distance summaries are reported in Supplementary Table S4 and Supplementary Dataset S1. With only three experimental batches, replicate was treated as a fixed factor rather than estimating a random-effect variance from three levels.

Gene-expression analyses were performed on individual ΔCt values. Because each of the eight plates contained all five experimental conditions and contributed one biological pool per treatment, plate was included as a blocking factor to account for any potential shared plate-level variation. For *th1*, *slc6a3*, *fosab*, *egr1*, *nr4a1*, *fkbp5*, and *kcnh2a*, fixed-block linear models of the form ΔCt = treatment + plate were fitted after checking residual normality. Treatment-versus-control contrasts were adjusted across the four planned contrasts for each gene using the Holm procedure. *npas4a* did not satisfy the parametric residual-normality criterion and was therefore analyzed using a Friedman test with plate as the block, followed by paired Wilcoxon signed-rank comparisons against the vehicle control with Holm adjustment. Stability of *ppiaa* was assessed using the same blocked linear-model framework. Statistical significance was set at *p* < 0.05.

## 5. Conclusions

Acute octodrine exposure affected neurobehavioral/transcriptional and cardiac endpoints over different concentration ranges under the conditions tested. Neurobehavioral effects and reduced expression of neuronal activity-responsive transcripts occurred at nanomolar-to-low-micromolar concentrations, whereas marked bradycardia and atrioventricular conduction impairment required substantially higher concentrations. Because the behavioural and cardiac assays used different concentration series with limited overlap, these findings should not be interpreted as defining sharply separated toxicological thresholds. The absence of significant changes in *th1*, together with only a modest overall effect on *slc6a3* that did not translate into significant treatment-versus-control differences, argues against major acute transcriptional remodelling of dopaminergic machinery, although non-transcriptional effects on monoaminergic signalling cannot be excluded. In parallel, *fkbp5* induction is consistent with engagement of glucocorticoid-responsive stress signalling. At higher concentrations, the cardiac phenotype indicates disruption of excitability and/or conduction, although its molecular basis remains unresolved. Collectively, these findings provide an initial zebrafish hazard characterization of octodrine and support targeted studies on monoaminergic signalling, glucocorticoid responses, internal dosimetry, and cardiac electrophysiology; direct extrapolation to human risk is not possible from nominal waterborne concentrations.

## Figures and Tables

**Figure 1:**
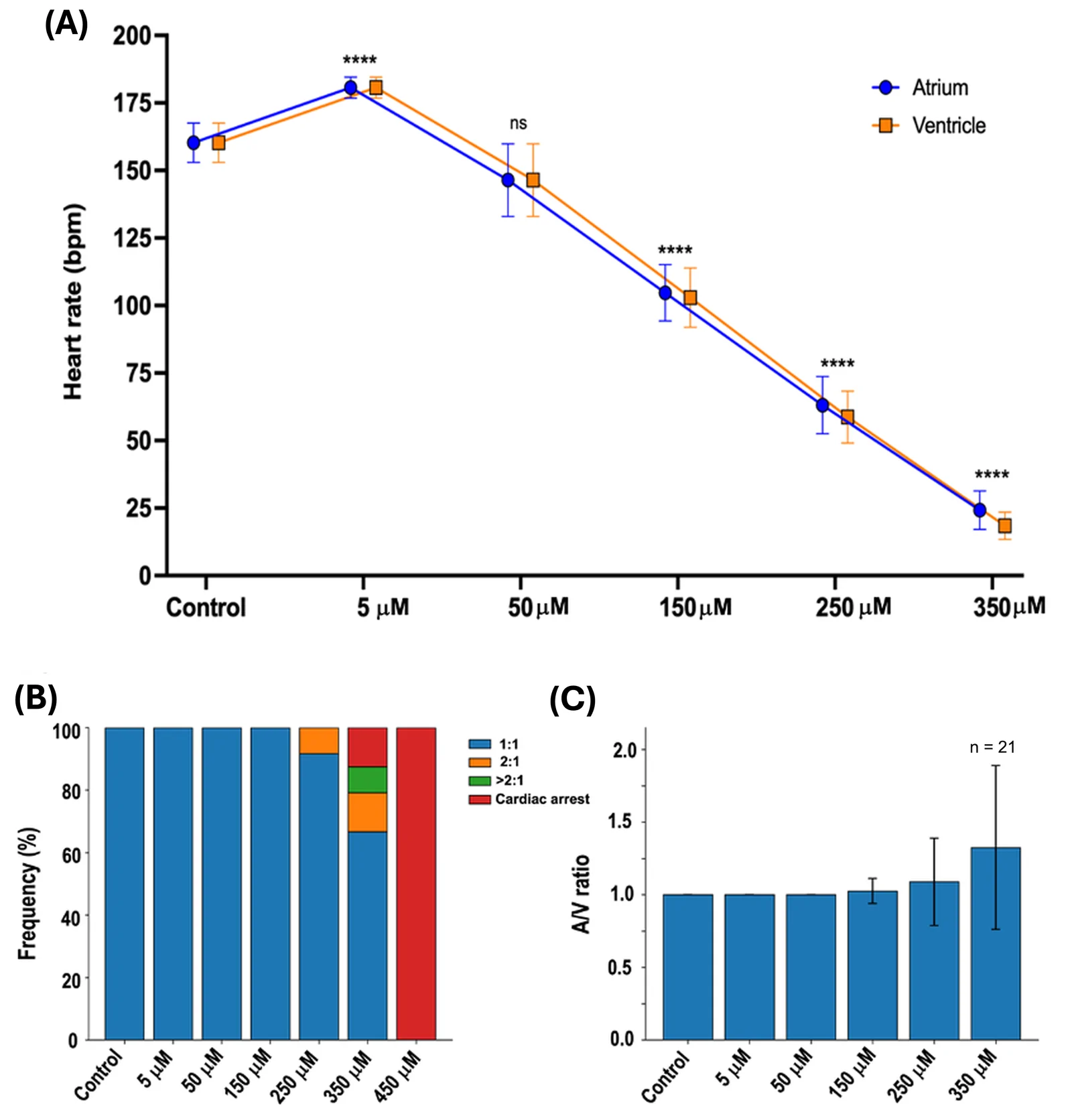
Cardiac effects of acute octodrine exposure in 7 dpf zebrafish larvae. (**A**) Atrial and ventricular heart rates, expressed as beats per minute (bpm), following 2 h of exposure to octodrine. Data are estimated marginal means with 95% confidence intervals from the GEE model. Asterisks indicate Holm-adjusted planned comparisons with the corresponding vehicle control within each chamber (**** *p* < 0.0001; ns, not significant). Because atrial and ventricular comparisons yielded the same significance category at each concentration, one annotation is shown per concentration. (**B**) Distribution of cardiac conduction/status categories (1:1, 2:1, >2:1, and cardiac arrest) following octodrine exposure. Ordinal logistic regression including experimental replicate as a covariate showed a significant concentration-dependent shift toward more severe categories (*p* < 0.0001). (**C**) Descriptive atrial-to-ventricular (A/V) ratio among larvae with detectable atrial and ventricular activity. Bars represent mean A/V ratios and error bars represent standard deviations (SD); no inferential test was applied to this bounded variable. For all groups, *n* = 24 larvae (eight per group in each of three independent experiments). At 350 µM, three cardiac-arrest larvae had undefined A/V ratios, leaving *n* = 21 for panel (**C**); 450 µM is absent from panels (**A**,**C**) because all larvae showed cardiac arrest.

**Figure 2:**
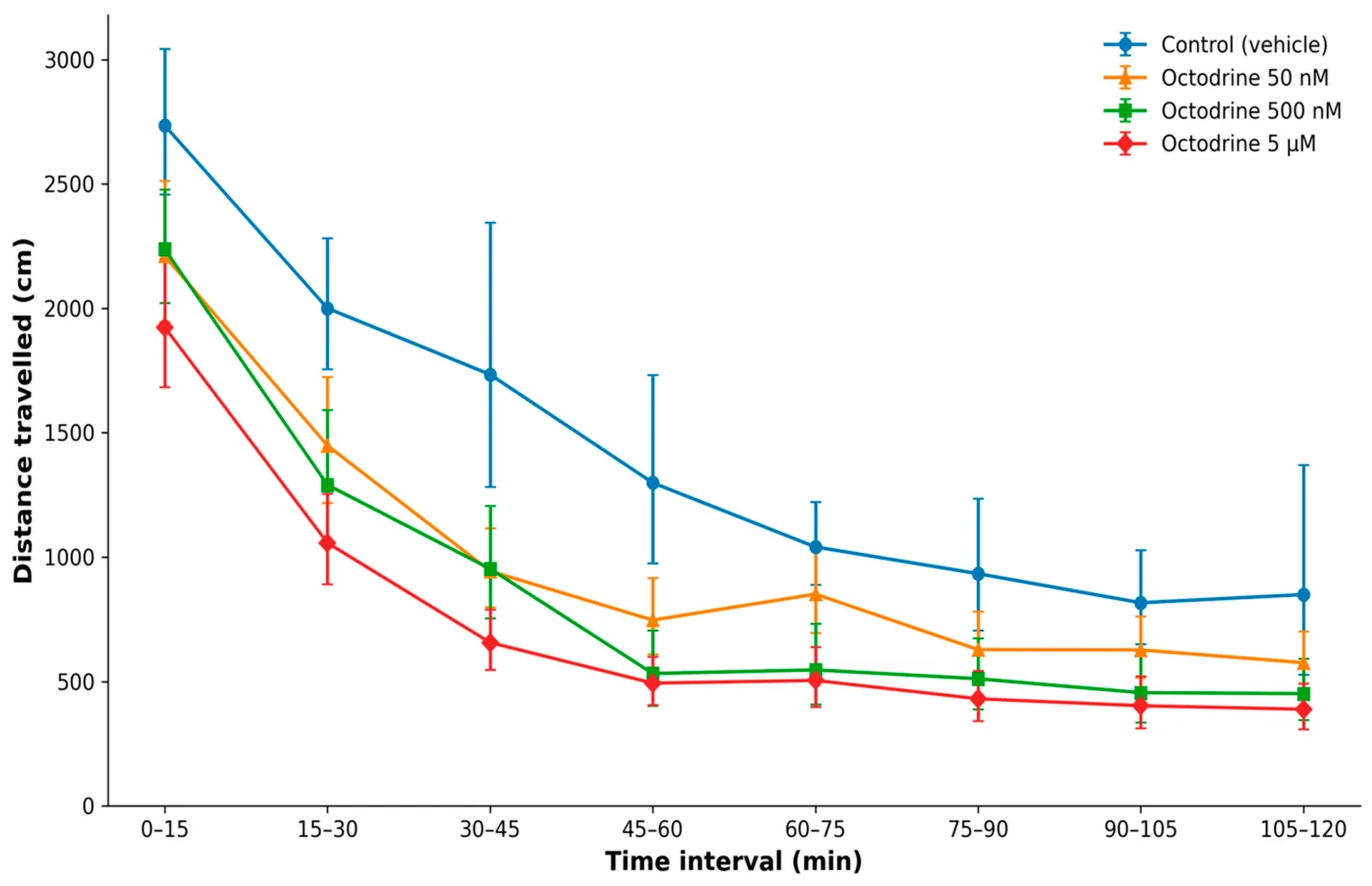
Effects of octodrine on spontaneous locomotor activity in zebrafish larvae. Locomotor activity was quantified as distance travelled during eight consecutive 15 min intervals over a 2 h exposure period (*n* = 36 larvae per group; 12 larvae per group in each of three independent experiments). Data are estimated marginal means with 95% confidence intervals from a GEE model using a gamma distribution with a log link, robust covariance estimation, and an AR(1) working correlation structure. To improve figure readability, significance annotations are not displayed in the panel. Planned treatment-versus-control comparisons were adjusted using a single sequential Bonferroni (Holm) procedure across all 24 time-by-treatment contrasts; complete raw and adjusted *p*-values are reported in Supplementary Table S4 and Supplementary Dataset S1.

**Figure 3:**
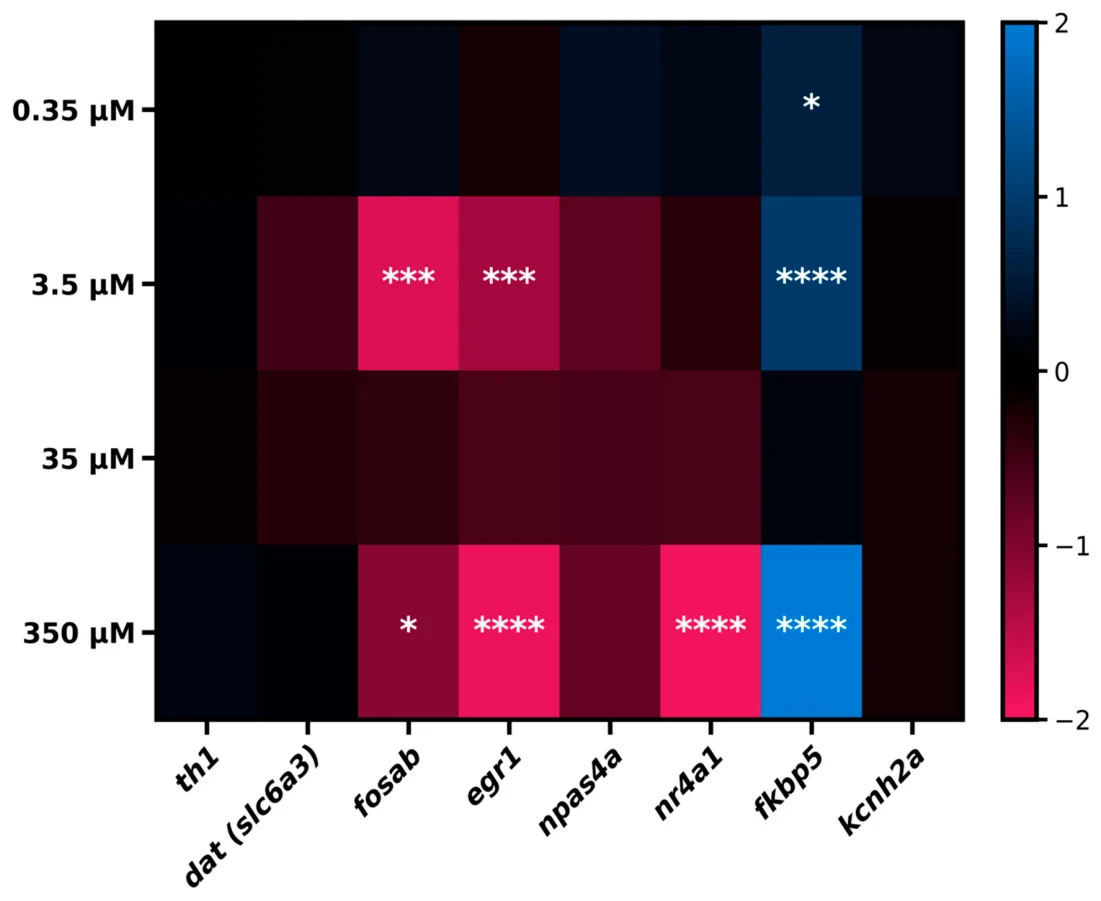
Relative expression of genes associated with dopaminergic function, neuronal activity, stress signalling, and cardiac electrophysiology following acute octodrine exposure. Heat map showing mean efficiency-corrected log_2_ fold changes (log_2_FC) in *th1*, *slc6a3* (DAT), *fosab*, *egr1*, *npas4a*, *nr4a1*, *fkbp5*, and *kcnh2a* after 2 h of exposure to 0.35–350 µM octodrine (*n* = 8 biological samples per group; each sample was a pool of six larvae, with one pool per condition from each of eight plates). Values are expressed relative to the vehicle control, which was set to log_2_FC = 0 and is therefore not displayed. Statistical inference was performed on individual ΔCt values. For *th1*, *slc6a3*, *fosab*, *egr1*, *nr4a1*, *fkbp5*, and *kcnh2a*, treatment and plate were included in fixed-block linear models and treatment-versus-control contrasts were Holm-adjusted. *npas4a* was analyzed using a Friedman test blocked by plate followed by paired Wilcoxon signed-rank comparisons with Holm adjustment. Asterisks indicate statistically significant adjusted treatment-versus-control differences (* *p* < 0.05, *** *p* < 0.001, **** *p* < 0.0001). Individual Cp and ΔCt values, efficiency-corrected expression values, and the full blocked analyses are provided in Supplementary Dataset S1.

## Data Availability

The original contributions presented in this study are included in the article/Supplementary Materials. Further inquiries can be directed to the corresponding authors.

## Supplementary Materials

The following supporting information can be downloaded at: https://www.mdpi.com/article/10.3390/ijms27198560/s1.
