# Supplementary material for: Acute Octodrine (DMHA) Exposure Disrupts Cardiac Function, Locomotor Activity, and Neuronal Activity-Responsive Transcription in Zebrafish Larvae
Source: Int J Mol Sci. 2026 Sep 24;27(19):8560. doi: 10.3390/ijms27198560 (PMC13665193; doi:10.3390/ijms27198560)
Supplement: Supplementary file 1 [file ijms-27-08560-s001.zip › SM_final_revised version.pdf]

Supplementary Materials

Acute Octodrine (DMHA) Exposure Disrupts Cardiac Function, Locomotor Activity, and Neuronal Activity-Responsive Transcription in Zebrafish Larvae

Eduardo Kennedy Carrão-Dantas, Ouwais Aljabasini, Eva Prats, Israel Felzenszwalb, Carlos Fernando Araujo-Lima and Demetrio Raldúa

Supplementary Tables

Supplementary Table S1. Generalized estimating equation analysis of atrial and ventricular heart rate following acute octodrine exposure.

A. Global GEE model effects

Effect	Wald χ^2	df	p
Concentration	2286.907	5	<0.0001
Cardiac chamber	9.814	1	0.0017
Experiment	3.607	2	0.1647
Concentration × cardiac chamber	12.390	3	0.0062

B. Planned simple contrasts: each octodrine concentration versus vehicle control

Chamber	Octodrine (µM)	Difference vs control (bpm)	SE	Wald χ^2	df	Holm-adjusted p	Significance
**Atrium**	5	20.386	4.213	23.416	1	<0.0001	****
**Atrium**	50	-13.851	7.802	3.151	1	0.0759	ns
**Atrium**	150	-55.550	6.488	73.317	1	<0.0001	****
**Atrium**	250	-97.190	6.562	219.356	1	<0.0001	****
**Atrium**	350	-136.046	5.198	685.031	1	<0.0001	****
**Ventricle**	5	20.386	4.213	23.416	1	<0.0001	****
**Ventricle**	50	-13.851	7.802	3.151	1	0.0759	ns
**Ventricle**	150	-57.341	6.735	72.490	1	<0.0001	****
**Ventricle**	250	-101.589	6.158	272.121	1	<0.0001	****
**Ventricle**	350	-141.799	4.519	984.713	1	<0.0001	****

C. Estimated marginal means from the GEE model

Chamber	Octodrine (µM)	Estimated mean (bpm)	SE	95% CI lower	95% CI upper
Atrium	0	160.278	3.723	152.982	167.575
Ventricle	0	160.278	3.723	152.982	167.575
Atrium	5	180.664	1.972	176.799	184.529
Ventricle	5	180.664	1.972	176.799	184.529
Atrium	50	146.427	6.857	132.988	159.867
Ventricle	50	146.427	6.857	132.988	159.867
Atrium	150	104.728	5.313	94.315	115.142
Ventricle	150	102.937	5.612	91.937	113.937
Atrium	250	63.089	5.404	52.497	73.680
Ventricle	250	58.689	4.906	49.074	68.304
Atrium	350	24.233	3.628	17.123	31.342
Ventricle	350	18.479	2.561	13.460	23.499

*Notes. GEE was fitted to raw heart-rate values (bpm) using a normal distribution with identity link, a robust covariance estimator, and an unstructured working correlation matrix. Larva was specified as the subject, with cardiac chamber (atrium/ventricle) as the within-subject factor. Octodrine concentration and cardiac chamber were included as fixed effects together with their interaction; experimental replicate was included as a blocking factor. The 450 µM group was excluded from quantitative heart-rate analysis because no atrial or ventricular activity was detectable in any of the 24 larvae during the standardized recording; these observations were operationally classified as cardiac arrest. Mortality was not independently assessed. Planned comparisons were made against the corresponding vehicle control within each chamber, with sequential Bonferroni (Holm) adjustment across the five comparisons per chamber. Positive contrast estimates indicate increased heart rate versus control. Working correlation between atrial and ventricular measurements = 0.995. Significance: **** $p < 0.0001$; *** $p < 0.001$; ** $p < 0.01$; * $p < 0.05$; ns, not significant.*

Supplementary Table S2. Inhibitory-phase concentration–response modelling of octodrine effects on heart rate.

Chamber	IC_{50} (µM)	95% CI	Hill slope	95% CI (Hill)	R^2	RMSE (% control)
Atrium	195.6	179.4–211.7	2.40	1.86–2.94	0.732	17.09
Ventricle	188.8	174.1–203.5	2.58	2.02–3.14	0.761	16.57

Notes. Heart-rate values were normalized to the mean vehicle-control value from the corresponding experimental replicate and cardiac chamber (vehicle = 100%). The inhibitory model was $Y = 100/[1 + (X/IC_{50})^h]$, *where h is the Hill slope, and was fitted separately for atrium and ventricle to 50, 150, 250, and 350 µM. The 5 µM group was excluded because it belongs to the initial stimulatory phase of the biphasic response. The 450 µM group was excluded because no cardiac activity was detectable in any larva.* IC_{50} *denotes the concentration producing 50% inhibition of heart rate relative to the experiment-specific vehicle control. Confidence intervals are Wald intervals from the nonlinear least-squares covariance matrix;* R^2 *and RMSE are provided as model diagnostics. Individual normalized values and residuals are available in Supplementary Dataset S1.*

Supplementary Table S3. Cardiac conduction and cardiac-status analysis following acute octodrine exposure.

A. Distribution of ordered cardiac-status categories

Octodrine	1:1	2:1	>2:1	Cardiac arrest	Total n
Control	24	0	0	0	24
5 μM	24	0	0	0	24
50 μM	24	0	0	0	24
150 μM	24	0	0	0	24
250 μM	22	2	0	0	24
350 μM	16	3	2	3	24
450 μM	0	0	0	24	24

B. Descriptive A/V ratios among larvae with measurable atrial and ventricular activity

Octodrine	Total n	n with defined A/V ratio	Mean A/V ratio	SD
Control	24	24	1.000	0.000
5 μM	24	24	1.000	0.000
50 μM	24	24	1.000	0.000
150 μM	24	24	1.026	0.087
250 μM	24	24	1.089	0.300
350 μM	24	21	1.325	0.564
450 μM	24	0	Undefined	—

C. Ordinal logistic regression of cardiac-status severity

Effect	β / coding	Odds ratio (95% CI)	Likelihood-ratio test
Octodrine concentration rank	β = 4.486; rank 0–6	88.74 (15.37–512.41)	$\chi^2(1) = 143.88$; $p < 0.0001$
Experimental replicate	2 indicator covariates	—	$\chi^2(2) = 5.84$; $p = 0.054$

D. Model diagnostics and sensitivity analyses

Analysis / check	Result	Interpretation
Primary ordinal-model convergence	Converged (BFGS; no optimizer warning)	Primary proportional-odds model was numerically stable.
Proportional-odds diagnostic	Threshold >1:1: β = 3.453, SE = 0.760; higher cumulative thresholds were non-estimable because severe outcomes were sparse and separated by concentration.	A formal Brant-type test was therefore not reliable for these data; robustness was assessed using models that do not depend on the separately plated 450 µM group.
Ordinal sensitivity excluding 450 µM	OR = 11.71 per rank step (95% CI: 2.39–57.32); LR $\chi^2(1) = 31.25$, $p < 0.0001$	Strong concentration trend persists after removing the group fully confounded with Plate 2.
Binary sensitivity excluding 450 µM	Any impairment vs 1:1: OR = 9.27 (95% CI: 2.08–41.24); $p = 0.0034$	Conclusion does not depend on the proportional-odds assumption.

Notes. For larvae with measurable atrial and ventricular activity, A/V = atrial rate/ventricular rate. A/V was not calculated when both chamber rates were zero. Cardiac status was classified as 1:1 (A/V < 1.5), 2:1 (1.5 ≤ A/V < 2.5), >2:1 (A/V ≥ 2.5), or cardiac arrest (atrial rate = 0 and ventricular rate = 0). Increasing category represented increasing cardiac/conduction impairment. The primary cumulative-logit model coded concentration as an ordered rank (0 = control, 1 = 5 µM, 2 = 50 µM, 3 = 150 µM, 4 = 250 µM, 5 = 350 µM, 6 = 450 µM) and included experimental replicate as indicator covariates. The 450 µM group occupied a separate plate within each experiment and was therefore completely confounded with plate identity; it was excluded in the sensitivity models shown in panel D. At 450 µM, absence of detectable cardiac activity was observed in 24/24 larvae; mortality was not independently assessed. Full individual classifications and model audit information are also provided in Supplementary Dataset S1.

Supplementary Table S4. Generalized estimating equation analysis of locomotor activity during acute octodrine exposure.

A. Global GEE model effects

Effect	Wald χ^2	df	*p*
Treatment	41.366	3	5.47E-09
Time interval	593.653	7	<0.0001
Experimental replicate	46.589	2	7.64E-11
Treatment × time interval	44.867	21	0.0018

B. Planned simple contrasts: each octodrine concentration versus vehicle control within each 15-min interval

Time interval	Octodrine	Difference vs control	SE	Wald χ^2	df	Holm-adjusted *p*	Significance
0–15 min	50 nM	-524.524	207.479	6.391	1	0.0803	ns
0–15 min	500 nM	-497.082	188.588	6.948	1	0.0715	ns
0–15 min	5 µM	-811.143	198.759	16.655	1	0.0009	***
15–30 min	50 nM	-551.741	185.966	8.802	1	0.0391	*
15–30 min	500 nM	-709.897	191.985	13.673	1	0.0035	**
15–30 min	5 µM	-943.085	162.853	33.536	1	<0.0001	****
30–45 min	50 nM	-790.206	278.928	8.026	1	0.0553	ns
30–45 min	500 nM	-780.829	290.321	7.234	1	0.0715	ns
30–45 min	5 µM	-1077.453	273.954	15.468	1	0.0016	**
45–60 min	50 nM	-552.023	205.798	7.195	1	0.0715	ns
45–60 min	500 nM	-766.615	205.202	13.957	1	0.0032	**
45–60 min	5 µM	-805.278	196.725	16.756	1	0.0009	***

Time interval	Octodrine	Difference vs control	SE	Wald χ²	df	Holm-adjusted *p*	Significance
60–75 min	50 nM	-189.539	122.361	2.399	1	0.2885	ns
60–75 min	500 nM	-494.223	117.413	17.718	1	0.0006	***
60–75 min	5 µM	-536.531	104.045	26.592	1	<0.0001	****
75–90 min	50 nM	-305.344	150.410	4.121	1	0.2118	ns
75–90 min	500 nM	-422.415	151.326	7.792	1	0.0577	ns
75–90 min	5 µM	-502.924	142.721	12.417	1	0.0064	**
90–105 min	50 nM	-189.636	113.977	2.768	1	0.2885	ns
90–105 min	500 nM	-361.345	118.951	9.228	1	0.0334	*
90–105 min	5 µM	-413.974	109.121	14.392	1	0.0027	**
105–120 min	50 nM	-273.965	215.255	1.620	1	0.2885	ns
105–120 min	500 nM	-398.329	216.532	3.384	1	0.2633	ns
105–120 min	5 µM	-461.103	212.493	4.709	1	0.1800	ns

C. Overall treatment effect within each time interval

Time interval	Wald χ²	df	p
0–15 min	16.836	3	7.64E-04
15–30 min	34.250	3	1.75E-07
30–45 min	22.504	3	5.12E-05
45–60 min	22.329	3	5.57E-05
60–75 min	32.990	3	3.24E-07
75–90 min	15.011	3	0.0018

Time interval	Wald χ²	df	p
90–105 min	18.313	3	3.79E-04
105–120 min	9.855	3	0.0198

D. Estimated marginal means from the final AR(1) GEE model

Time interval	Treatment	Estimated mean	SE	95% CI lower	95% CI upper
0–15 min	Vehicle control	2734.612	148.774	2458.028	3042.318
0–15 min	50 nM	2210.088	144.616	1944.070	2512.508
0–15 min	500 nM	2237.530	115.894	2021.530	2476.609
0–15 min	5 µM	1923.469	131.801	1681.740	2199.943
15–30 min	Vehicle control	2000.108	133.978	1754.025	2280.717
15–30 min	50 nM	1448.368	128.971	1216.418	1724.546
15–30 min	500 nM	1290.212	137.508	1046.989	1589.937
15–30 min	5 µM	1057.024	92.581	890.289	1254.985
30–45 min	Vehicle control	1733.976	266.973	1282.292	2344.764
30–45 min	50 nM	943.770	80.786	798.002	1116.165
30–45 min	500 nM	953.147	114.069	753.861	1205.115
30–45 min	5 µM	656.523	61.450	546.485	788.718
45–60 min	Vehicle control	1298.656	190.532	974.116	1731.321
45–60 min	50 nM	746.633	77.785	608.734	915.770
45–60 min	500 nM	532.041	76.193	401.833	704.441
45–60 min	5 µM	493.378	48.974	406.151	599.339

Time interval	Treatment	Estimated mean	SE	95% CI lower	95% CI upper
60–75 min	Vehicle control	1040.752	84.407	887.796	1220.061
60–75 min	50 nM	851.214	88.587	694.150	1043.816
60–75 min	500 nM	546.529	81.617	407.848	732.367
60–75 min	5 µM	504.221	60.835	398.036	638.733
75–90 min	Vehicle control	933.370	133.282	705.513	1234.818
75–90 min	50 nM	628.026	69.707	505.242	780.650
75–90 min	500 nM	510.955	71.661	388.153	672.609
75–90 min	5 µM	430.446	51.039	341.184	543.060
90–105 min	Vehicle control	816.413	95.393	649.310	1026.521
90–105 min	50 nM	626.777	62.377	515.705	761.773
90–105 min	500 nM	455.069	71.060	335.089	618.008
90–105 min	5 µM	402.439	52.986	310.906	520.920
105–120 min	Vehicle control	849.433	207.351	526.437	1370.603
105–120 min	50 nM	575.468	57.797	472.641	700.666
105–120 min	500 nM	451.104	62.384	344.004	591.549
105–120 min	5 µM	388.331	46.464	307.153	490.962

E. Cumulative 2 h distance travelled in each independent experiment

Experiment	Treatment	n larvae	Mean cumulative distance (cm)	SD	% of experiment control
1	Control	12	19695.8	13959.6	100.0
1	50 nM	12	9171.5	1925.5	46.6
1	500 nM	12	12407.9	5618.8	63.0
1	5 µM	12	7554.5	2465.6	38.4
2	Control	12	9997.3	2004.3	100.0
2	50 nM	12	8212.7	2020.9	82.1
2	500 nM	12	5451.6	1436.6	54.5
2	5 µM	12	6038.3	1784.1	60.4
3	Control	12	7449.0	2571.8	100.0
3	50 nM	12	6478.4	2714.5	87.0
3	500 nM	12	4935.0	1786.8	66.3
3	5 µM	12	4275.1	1673.8	57.4

F. Sensitivity analysis of treatment-by-experimental-replicate heterogeneity

Effect	Wald χ^2	df	*p*	Interpretation
Treatment × experimental replicate	27.573	6	0.000113	Effect magnitude differed among experiments

G. Experiment-specific model-derived treatment/control ratios from the expanded sensitivity GEE

Experiment	Treatment	Estimated % of experiment-specific control	95% CI
1	50 nM	44.3	28.2–69.8
1	500 nM	66.5	40.1–110.4
1	5 µM	32.5	20.2–52.2
2	50 nM	78.6	64.8–95.5
2	500 nM	52.2	42.5–64.2
2	5 µM	58.7	47.8–72.1
3	50 nM	95.2	70.7–128.2

3	500 nM	66.0	50.1–87.1
3	5 µM	70.4	52.3–94.8

Notes. Locomotor activity was analyzed using generalized estimating equations (GEE) applied to the raw distance-travelled data, with a gamma distribution and log link. Larva identity was specified as the subject and time interval as the within-subject repeated factor. Treatment, time interval, and treatment × time interval were included as fixed effects; experimental replicate was included as a blocking factor. A robust sandwich covariance estimator and an AR(1) working correlation structure were used. The estimated adjacent-interval correlation was $\rho = 0.563$. Model fit: QIC = 654.788; QICC = 636.326. Estimated marginal means are shown on the original response scale. Planned comparisons are treatment-minus-control differences within each time interval; negative values indicate reduced locomotor activity relative to vehicle. To control the family-wise error rate, p values were Holm-adjusted across all 24 treatment-versus-control contrasts. Cumulative distance in panel E was calculated as the sum of the eight 15-min distance values for each larva. The expanded sensitivity GEE in panel F added a treatment × experimental-replicate interaction (Wald $\chi^2(6) = 27.573$, $p = 0.000113$). Panel G reports model-derived ratios obtained by summing the eight predicted interval means for each treatment and dividing by the corresponding predicted control sum within each experiment, with delta-method 95% CIs calculated on the log-ratio scale. These ratios are sensitivity/reproducibility estimates rather than additional primary multiplicity-adjusted tests. The 5 µM 95% CI remained below 100% in all three experiments.

Supplementary Table S5. Statistical analysis of qRT-PCR gene-expression data following acute octodrine exposure.

A. Global tests and assumption checks

Gene	Primary analysis	Residual Shapiro-Wilk p	Treatment statistic	Treatment p	Plate p	Post hoc
th1	Blocked linear model	0.183	F(4,28) = 0.879	0.489	0.514	Holm vs control
slc6a3	Blocked linear model	0.612	F(4,28) = 3.563	0.0180	0.197	Holm vs control
fosab	Blocked linear model	0.672	F(4,28) = 8.600	0.000117	0.252	Holm vs control
egr1	Blocked linear model	0.847	F(4,28) = 15.430	<0.0001	0.103	Holm vs control
npas4a	Friedman blocked by plate	—	Q(4) = 16.300	0.00264	Blocked by design	Paired Wilcoxon-Holm
nr4a1	Blocked linear model	0.208	F(4,28) = 25.059	<0.0001	0.595	Holm vs control
fkbp5	Blocked linear model	0.148	F(4,28) = 33.121	<0.0001	0.174	Holm vs control
kcnh2a	Blocked linear model	0.557	F(4,28) = 2.173	0.0980	0.840	Holm vs control

B. Adjusted post hoc comparisons with the vehicle control

Gene	0.35 µM vs control	3.5 µM vs control	35 µM vs control	350 µM vs control
th1	1.000 (ns)	1.000 (ns)	1.000 (ns)	1.000 (ns)
slc6a3	1.000 (ns)	0.0844 (ns)	0.4308 (ns)	1.000 (ns)

Gene	0.35 µM vs control	3.5 µM vs control	35 µM vs control	350 µM vs control
fosab	0.6295 (ns)	0.000538 (***)	0.6295 (ns)	0.0315 (*)
egr1	0.4578 (ns)	0.000223 (***)	0.0833 (ns)	1.22×10^{-6} (****)
npas4a	0.4453 (ns)	0.4453 (ns)	0.4453 (ns)	0.1563 (ns)
nr4a1	0.3323 (ns)	0.3323 (ns)	0.0524 (ns)	4.31×10^{-8} (****)
fkbp5	0.0134 (*)	9.28E-05 (****)	0.3524 (ns)	2.25×10^{-10} (****)
kcnh2a	0.7589 (ns)	0.7589 (ns)	0.7589 (ns)	0.7589 (ns)

*Notes. The qRT-PCR experiment comprised one exposure run using eight plates, with one pool of six larvae per treatment obtained from each plate. Vehicle control, 0.35, 3.5, 35, and 350 µM occupied rows A–E (wells 1–6), respectively, and these positions were kept constant across plates. Statistical inference was performed on individual ΔCt values with plate incorporated as a blocking factor. For th1, slc6a3, fosab, egr1, nr4a1, fkbp5, and kcnh2a, fixed-block linear models (ΔCt = treatment + plate) were used after checking residual normality; treatment-versus-control contrasts were Holm-adjusted across the four planned contrasts per gene. npas4a did not meet the residual-normality criterion and was analysed using a Friedman test blocked by plate followed by paired Wilcoxon signed-rank comparisons with Holm adjustment. The reference gene ppiaa was stable in the same blocked framework (treatment: $F(4,28) = 0.323$, $p = 0.860$; plate: $F(7,28) = 0.595$, $p = 0.754$; residual Shapiro-Wilk $p = 0.599$). Values in panel B are multiplicity-adjusted p values. Significance: **** $p < 0.0001$; *** $p < 0.001$; ** $p < 0.01$; * $p < 0.05$; ns, not significant. Individual Cp and ΔCt values, efficiency-corrected expression values, and the complete blocked-analysis audit are provided in Supplementary Dataset S1.*

Supplementary Table S6. List of primers used for the qPCR.

Gene	ZFIN Acc number	GenBank Acc number		Sequence	Amplicon length
egr1	ZDB-GENE-980526-320	NM_131248	FW	5'-TAAGATCCACATGCGGCAGA	118 bp
			RV	5'-AGAGGGGTAGCTGGACACTG	
fkbp5	ZDB-GENE-030616-630	NM_213149.2	FW	5'-TCAACCTGCAAACCCTGGTA	135 bp
			RV	5'-AACGAACAAGCGGGTCTGAA	
fosab	ZDB-GENE-031222-4	NM_205569	FW	5'-ATGGCATTGCAGGGCTATGG	80 bp
			RV	5'-GGCAGGCATGTATGGTTCAGA	
kcnh2a	ZDB-GENE-070912-699	NM_001042722.2	FW	5'-ACCCACACTTGTGATGTTGTC	81 bp
			RV	5'-TGGCATACATACTTGAAGCCT	
npas4a	ZDB-GENE-060616-396	NM_001045321	FW	5'-AGCAGGAAAAATAGGGACAGGG	96 bp
			RV	5'-ACGTTAGCATGTCGACACCA	
nr4a1	ZDB-GENE-040704-11	NM_001002173.1	FW	5'-GGTGTGAGATCCAGTGCAGC	94 bp
			RV	5'-GGTACCGCACACTAAGCCAG	
ppiaa	ZDB-GENE-030131-8556	NM_212758.2	FW	5'-TTGTGGAGGGCTTGGATGTC	169 bp
			RV	5'-GGCAGGATTTCGGAAAGGGA	
slc6a3	ZDB-GENE-010316-1	NM_131755.1	FW	5'-GCCTGGTTTTACGGAGTGGA	110 bp
			RV	5'-AGGAAACACGGGCTCACAAA	
th1	ZDB-GENE-990621-5	NM_131149.1	FW	5'-GCTTTGTGGACGCTACTGAG	72 bp
			RV	5'-CGAACCGCACAGAAAACGG	

Supplementary Figures

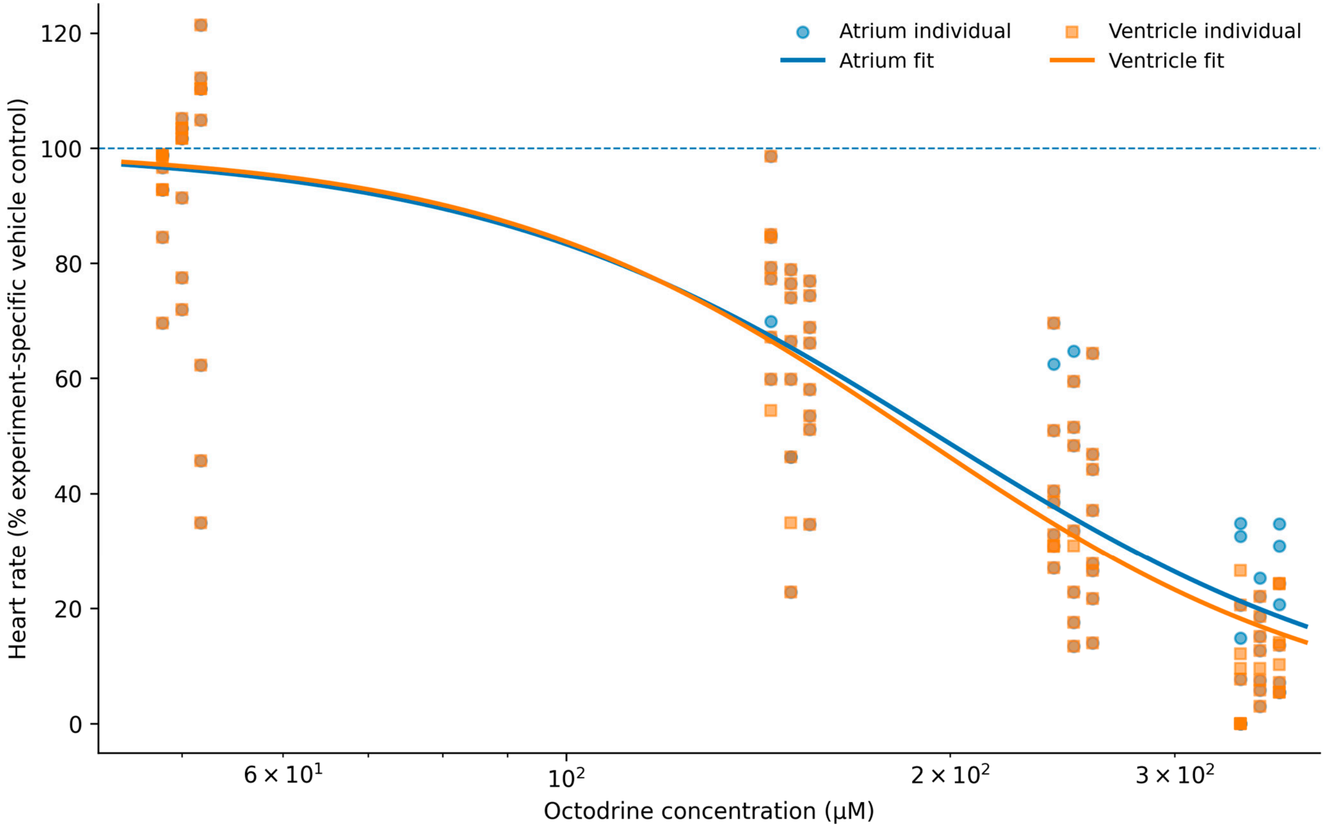

Supplementary Figure S1. **Inhibitory-phase concentration–response modelling of atrial and ventricular heart rate following acute octodrine exposure.** Individual heart-rate observations at 50, 150, 250, and 350 µM octodrine are expressed as percentages of the corresponding experiment-specific vehicle-control mean (vehicle = 100%) and are shown together with the fitted inhibitory concentration–response curves for atrial and ventricular rate. Curves were fitted according to $Y = 100/[1 + (X/IC_{50})^h]$. The 5 µM group was excluded because it belonged to the initial stimulatory phase of the biphasic cardiac response, whereas the 450 µM group was excluded because no detectable cardiac activity was observed in any larva. Slight horizontal offsets were applied to overlapping observations to distinguish experimental replicates.

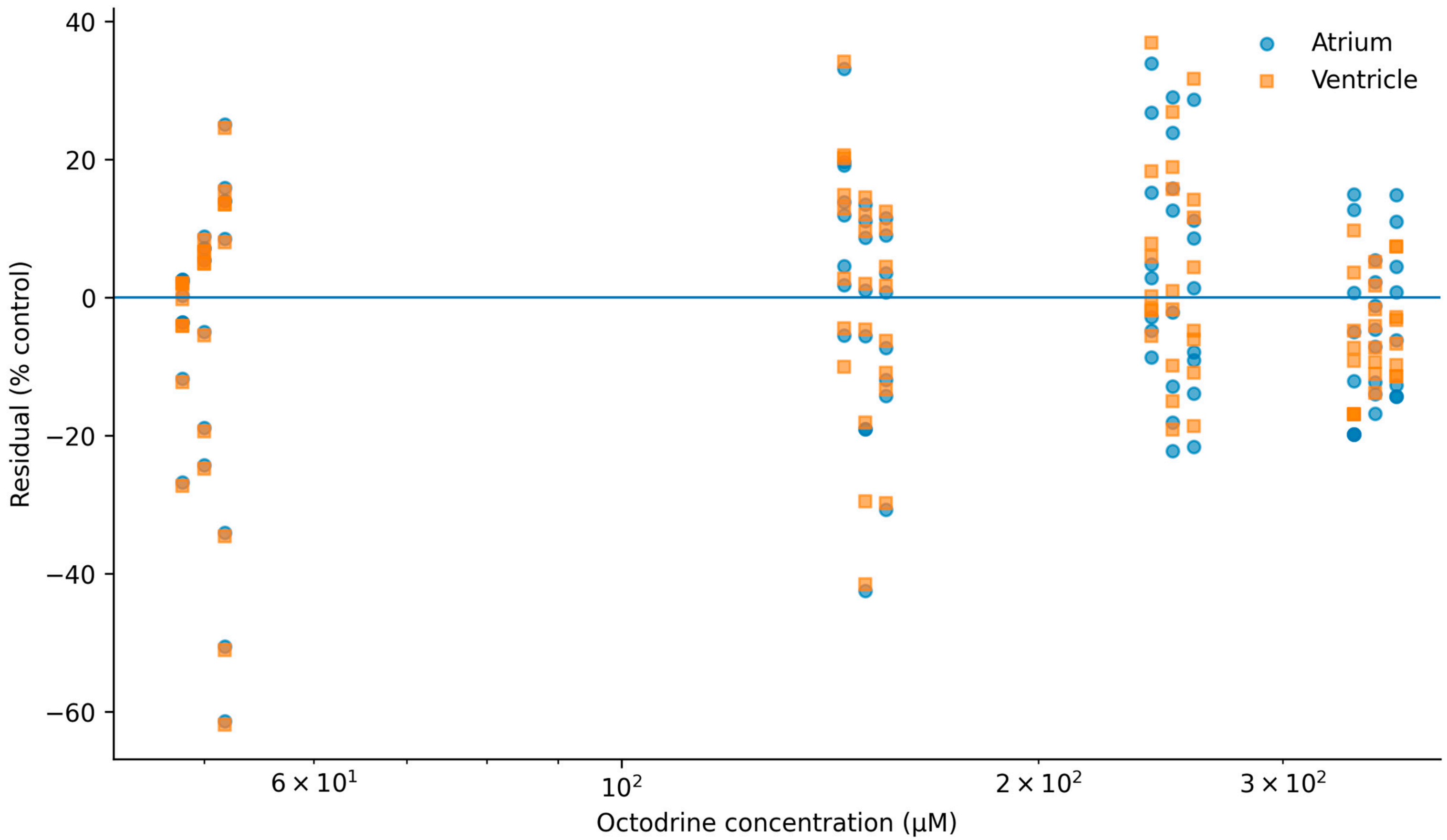

Supplementary Figure S2. Residual diagnostics for the inhibitory-phase cardiac concentration–response models. Individual residuals, calculated as observed normalized heart rate minus model-predicted response, are shown for the atrial and ventricular fits at 50, 150, 250, and 350 μM octodrine. The horizontal dashed line represents a residual of zero. Slight horizontal offsets were applied to overlapping observations to distinguish experimental replicates.
